# Small-scale urban agriculture: Drivers of growing produce at home and in community gardens in Detroit

**DOI:** 10.1371/journal.pone.0256913

**Published:** 2021-09-07

**Authors:** Carola Grebitus

**Affiliations:** W. P. Carey School of Business, Morrison School of Agribusiness, Arizona State University, Mesa, Arizona, United States of America; University of Florida, UNITED STATES

## Abstract

The desire for fresh, local food has increased interest in alternative food production approaches, such as private small-scale agriculture, wherein households grow their own food. Accordingly, it is worth investigating private agricultural production, especially in urban areas, given that an increasing share of the world’s population is living in cities. This study analyzed the growth of produce at people’s homes and in community gardens, focusing on behavioral and socio-demographic factors. Data were collected through an online survey in Detroit, Michigan; 420 citizens were interviewed. The results revealed that trust, attitude, and knowledge affect the growing of produce at home. Involvement and personality are also drivers of community gardening. Regarding socio-demographics, household size affects the growing of produce at home, while gender, age, and income affect community gardening. The findings have valuable implications for stakeholders who wish to foster private small-scale urban agriculture, for example, through city planning and nutrition education.

## 1. Introduction

In 2018, approximately 55% of the world population lived in urban areas, a number projected to rise to approximately 60% by 2030 [1]. This ratio is even higher for the United States, at 82% in 2018 [2]. This raises the question of how to nourish these residents with fresh and local foods [3].

Food production can be integrated into urban communities through commercial urban agriculture, private gardening (in yards, on balconies, or indoors), or community gardens [4, 5]. Urban agriculture for food production at household level, whether at home or in community gardens, is called “small-scale urban agriculture” [6]. In 2013, fully 35% of all 42 million US households were food gardening [7], a number that has likely grown. Hence, this study develops a conceptual framework describing behavioral and socio-demographic drivers of private fruit and vegetable gardening in urban areas in the U.S. focusing on Detroit. As the U.S. is diverse in temperature, precipitation, population, and many other factors, the findings will serve as a case study and are not generalizable nationwide.

Urban agriculture benefits communities and the local ecological system. Hendrickson and Porth [8] found that urban gardeners benefited from food production by supplementing household foods, bringing in cash, and enhancing the image of their neighborhood. Specht et al. [9] and Thomaier et al. [10] pointed out that urban agriculture supports basic food needs, offers social benefits, and provides an opportunity to educate consumers about food production. Reynolds and Cohen [11] discussed the role that urban agriculture plays in social justice. In addition, community gardening builds social capital and empowers people [12, 13], and gardening together fosters community cohesion, increasing social health [14, 15]. Stress reduction, positive emotions, social integration, and restored attention are other benefits of community gardens, ameliorating mental, social, and physical health [15]. Finally, Parece and Campbell [16] found that urban agriculture can positively impact the physical urban landscape and contribute to ecosystem services. All these benefits often depend on conscious efforts by farmers, and urban farms may include social components such as food security, community building, and education in their mission [17].

However, urban agriculture also involves challenges or detriments [18], for instance competition for resources, such as land, soil, and water [19], high investment costs or lack of acceptance [9], lack of space for large-scale urban farms [20], fostering of political and social inequity and displacement of urban neighbors [21], and soil contaminants that pose potential health risks [8, 22].

Given these advantages and disadvantages, identifying behavioral drivers can make gardening efforts more effective, and thus facilitate reaping the benefits while tackling the barriers. Information about key factors also helps stakeholders assist citizens in growing food, for example, providing information on use of fertilizers and pesticides to prevent runoff or ensuring that soils of vacant land are tested before allowing food production. Overall, uptake of food gardening is valuable for individuals and society if challenges are addressed.

This study investigates behavioral and socio-demographic drivers related to two forms of private small-scale urban agriculture: gardening at home and in community gardens. For instance, individuals who hold positive attitudes toward growing food are likely more disposed to participate in small-scale urban agriculture, at home or in a community garden. According to Grebitus et al. [3] and Alemu and Grebitus [23], subjective knowledge also affects participation in urban agriculture: To start growing food, participants require gardening knowledge including appropriate use of fertilizers and when to plant crops. Fellow gardeners at a community garden could offer insights, making less knowledgeable gardeners more likely to grow produce in the community garden. Furthermore, involvement affects decision-making [24], and trust impacts behavior [25]. Teig et al. [26] found that mutual trust was regarded as an asset by community gardeners, meaning those who are more trusting might be more likely not only to grow produce but also to do so in community gardens rather than at home. Finally, personality is a major predictor of behavior [27] and thus may also affect the growth of urban food. For instance, extraverts might be more likely to grow food in community gardens and introverts at home.

This study constructs a model incorporating these behavioral drivers of growing produce at home and in community gardens in urban areas. Complementing White [28], Colasanti et al. [29], and Pothukuchi [30], who qualitatively investigated urban gardening in Detroit, quantitative data in Detroit were collected via online survey. Detroit was chosen as the research site because of its prevalent food deserts and history of food access issues [31, 32], which might be alleviated by promoting small-scale urban agriculture. Furthermore, Detroit has exhibited rapid economic development and opportunities for local agriculture recently [33]. Detroit planners have started to incorporate urban gardens: about 1,400 urban gardens and farms out of approximately 52,000 farms total in Michigan in 2015 [34], and about 1,600 in 2019, engaging more than 25,000 residents [35].

Next, the literature on growing food in urban areas of developed countries is discussed before describing conceptual and methodological background, empirical results and deriving conclusions.

## 2. Literature

### 2.1 Community gardening

Several studies have examined the benefits of small-scale agriculture for food production, food security, and dietary patterns. Libman [36] conducted a study at the Brooklyn Botanic Garden (BBG) Children’s Garden, and found that growing food naturally increases knowledge of how to produce and process food and also increases consumption of produce. Similar results were found by Alaimo et al. [37], in a quantitative study in Flint, Michigan. Armstrong [12] found that the main reason for gardens in upstate New York is access to fresh foods, nature, and health benefits. Moreover, community gardens can empower the community and catalyze tackling of neighborhood issues, in turn improving public health. Wakefield et al. [14] studied community gardens in Toronto and found that gardeners are more physically active, with better mental health, improved food access, and nutrition. Additionally, community gardens enable food gardening by those who could not otherwise access it [14].

Wakefield et al. [14] stressed promotion of social health through the improvement of social cohesion. In the UK, Firth and colleagues [13], in Nottingham, pointed out that community gardens build social capital and empower members by linking them to institutions and authorities; Jackson [38] also found gardens created social capital in Lincoln. Alaimo et al. [39], in Flint, suggested that community gardeners’ involvement in activities and meetings is related to perceptions of bonding social capital.

Colasanti et al. [29], in Detroit, found a broad range of views on urban agriculture among its practitioners, from people envisioning agrarian cities to concern with food security, sustainability, and opportunities for poor economies. White [28] interviewed black female farmers in Detroit and found that urban gardening makes them a “change agent in their community” while producing healthy food for themselves and their community. Alemu and Grebitus [23] studied consumers’ preferences on community garden characteristics in Detroit and Phoenix (Arizona) and found that guidance regarding gardening and provision of tools were key considerations for participation.

### 2.2 Home gardening

Previous literature on the benefits of home gardening has established findings similar to those for community gardens. Taylor and Taylor Lovell [40, 41] studied home-gardening households in Chicago and found home gardening was beneficial for household food budgets, community food systems, urban agrobiodiversity, and cultural identity. A study in Toronto found that home gardening provided access to affordable and nutritious produce, aided community food security, improved health and well-being, and contributed to environmental sustainability, self-reliance, and cultural acceptability [42]. Sanye-Mengual et al. [43] assessed the eco-efficiency and food security potential of home gardens in Padua (Italy). In New Zealand, Van Lier et al. [44] analyzed adolescents and found that home gardening was positively related with consumption of produce, positive effects on social health, greater physical activity, and better mental health and well-being. Algert et al. [45] concluded that both community and home gardens can assist food security, in San Jose, California.

Overall, the studies on home gardening largely show the same benefits as for community gardening. Moreover, Taylor and Taylor Lovell [46], in Chicago, found that only a small percentage of garden sites were community gardens for producing food; home gardens accounted for most urban food production areas. They stated that home gardening has been understudied even though it “may make a far greater contribution to urban food systems than other forms of urban agriculture such as community gardens and urban farms” [41] (p. 301).

### 2.3 Deriving research questions

Although previous studies on small-scale urban agriculture have highlighted many benefits, they have largely focused on extrinsic motivations for participation and on opportunities and challenges for small-scale gardeners. Hence, this study investigates intrinsic and behavioral drivers of participation in small-scale urban agriculture. In response to Taylor and Taylor Lovell’s [41] claim that home gardens are not receiving enough research attention, both home and community gardening are considered. I investigate the following research questions:

1. What are the behavioral (psychological) characteristics of those who grow produce in urban settings, that is, at home or in community gardens?
2. What are the socio-demographic characteristics of those who grow produce in urban settings?

## 3. Conceptual framework

Grebitus et al. [3] investigated success factors of commercial urban agriculture, testing the influence of consumer perceptions, knowledge, attitude, and socio-demographics on the intention to purchase or grow produce at a commercial urban farm. This study extends their work by investigating the effects of knowledge, attitude, trust, involvement, personality, and socio-demographics on the growing of produce in urban settings.

### 3.1 Trust

Trust is based on an individual’s worldviews and moral values [47–49] and plays a role in food-related behavior, such as food safety and purchase of genetically modified food [25, 50, 51]. By interviewing garden leaders and community gardeners in Denver (Colorado), Teig et al. [26] found mutual trust was important among gardeners. Gardeners felt safe and comfortable inside the garden and mentioned that trust extended by fellow gardeners to themselves increased their own perceived importance. However, they also stated they did not trust people outside this circle and that visitors could trample the garden [26]. Accordingly, I tested whether growing produce in the community garden requires more trust than gardening at home.

### 3.2 Knowledge and attitude

Grebitus et al. [3] found a significant influence of subjective knowledge on behavior related to commercial urban agriculture. Kopiyawattage et al. [52] concluded that knowledge and skills affected perceived behavioral control, which in turn determined the decision to continue farming in urban areas. Moreover, the importance of personal knowledge of how food is grown was highlighted by Kortright and Wakefield [42], for home gardeners, and by Landry et al. [53], who described gardening as an educational tool capable of increasing self-efficacy and responsibility for health. Alemu and Grebitus [23] showed that proponents of growing food in community gardens are characterized by high subjective knowledge and opponents by low knowledge. Grebitus et al. [3] found that a positive attitude toward urban agriculture increased likelihood of purchasing or growing produce in a commercial urban agriculture setting. Similarly, Alemu and Grebitus [23] found that positive attitude shaped preference for community gardening for proponents of growing food and other consumer groups. However, the impact of attitudes differed between Phoenix and Detroit. Kopiyawattage et al. [52] found that for commercial urban food producers in Columbus (Ohio), positive attitudes significantly affected whether to continue farming. I tested whether the effects of attitude and knowledge differ between growing produce at home or in community gardens.

### 3.3 Involvement

Involvement is described as perceived personal relevance [54, 55]. Degree of involvement affects behavior [56], such as consumption patterns, food choice, and purchase decision-making [57, 58]. More involved consumers display more heterogeneous preferences for organic food [59] and more environmentally involved consumers use more product characteristics to make a purchase decision for organic milk [60]. Alaimo et al. [39] found that household involvement in community gardening was related to perception of links between social capital and a neighborhood’s norms and values. Given the complexity of growing produce, I tested whether individuals who are more involved are more likely to grow produce and whether this differs by setting.

### 3.4 Personality

Personality can provide information on behavior [27]. For instance, persons high in the personality trait *agency* are extremely assertive, self-confident, and outspoken. *Agreeableness* represents kindness, likeability, trustworthiness, and cooperativeness. *Conscientiousness* represents reliability, impulse control, responsibility, and willingness to work hard. *Extraverts* like to interact with others and are lively, active, and outgoing. An *open* person is creative, prefers novelty, and is flexible. *Neuroticism* describes anxiety, emotional instability, and sadness [61–64].

Grebitus et al. [65] found that higher extraversion was related to willingness to pay more for food, and Grebitus and Dumortier [66] showed that openness, extraversion, and conscientiousness affect demand for tomatoes. Lin et al. [67] found that openness and conscientiousness explain consumer acceptance of genetically modified (GM) meat. I investigated personality as a driver of growing produce at home or in community gardens, testing whether more open and conscientious (neurotic) persons are more (less) likely to grow produce, and whether extraverts and agreeable persons are more likely to do so in community gardens.

### 3.5. Conceptual framework for socio-behavioral drivers of home and community gardening

**Fig 1:**
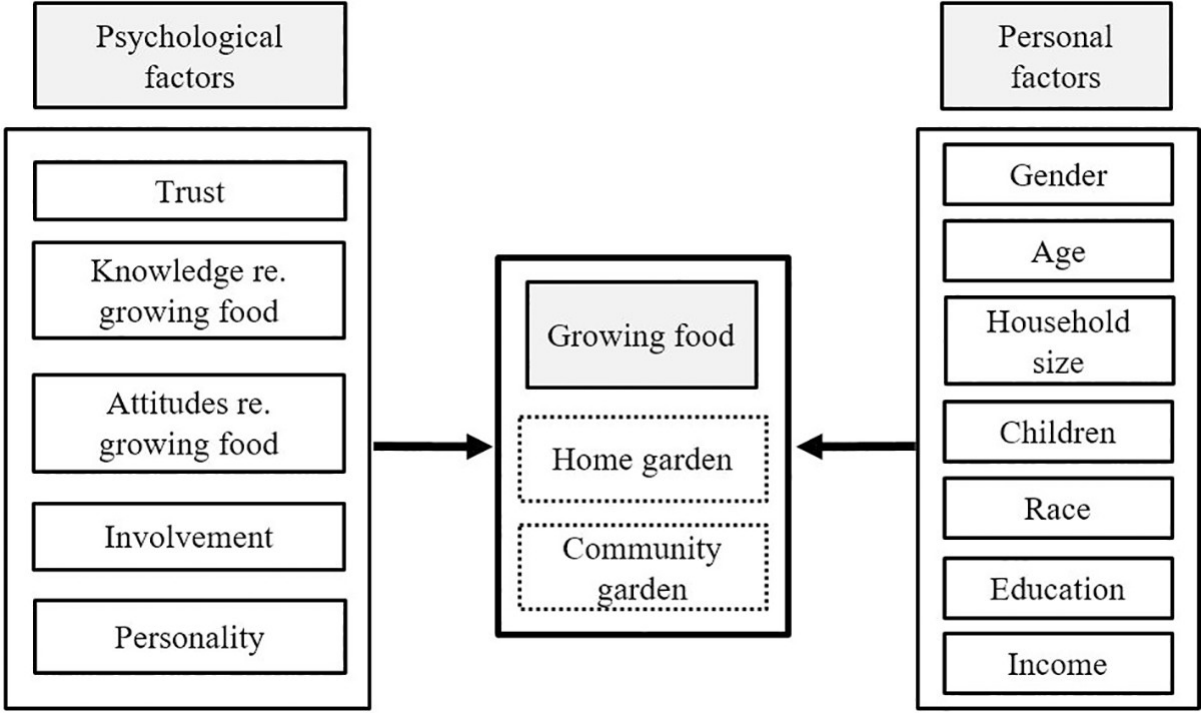
Conceptual framework: Socio-behavioral factors of participation in small-scale urban agriculture.

Based on the theoretical and empirical evidence, Fig 1 displays the conceptual framework, which provides a foundation to test socio-behavioral factors affecting the growing of produce at home and in community gardens. The influence of behavioral (psychological) constructs such as trust, knowledge, attitude, involvement, and personality on participation in small-scale urban agriculture was analyzed. In addition, socio-demographic (personal) factors were assessed, given that previous studies have found them to be drivers of urban gardening [37, 39, 68–70].

## 4. Methodological background

### 4.1 Data collection and sample characteristics

An online survey was used to collect data. The Arizona State University Institutional Review Board approved the survey (IRB ID: STUDY00005935), which was considered exempt research. At the beginning of the survey was stated: “Filling out the questionnaire will be considered your consent to participate”; consent was handled accordingly. Participants were recruited by the Qualtrics company based on residence in urban or suburban Detroit. Qualtrics recruits participants by aggregating online panel resources, where respondents are invited to a dashboard through an app or email notification to take the survey. Participants were reimbursed by Qualtrics either monetarily or in points redeemable for discounts, items, or money.

The survey was coded using the Qualtrics platform. Data were collected in Spring 2017 from 420 Detroiters. The same data were used by Alemu and Grebitus [23] and Chenarides et al. [70]. Qualtrics recruited a sample broadly matching Detroit’s socio-demographics in gender and age. The sample consisted of 50% female respondents (where Detroit is 53% female [71]), median age was 45 years old (*M* = 45, *SD* = 16.4), ranging from 18 to 88 years, which is older than Detroit’s median age of 35 years [72]. Education ranged from high school diploma (23%) to some college experience (27%), 2-year degree (10%), 4-year degree (26%), professional degree (9%), and doctorate (2%), while 2% had less than a high school degree; in Detroit, 80% are high school graduates and 15% hold a bachelor’s degree (or higher) [71]. However, the sample is not representative of race; 15% of Detroiters are White, but White participants comprised 74% of the research sample. Approximately 50% of interviewees had income lower than $50,000 annually before taxes, whereas median household income in Detroit is $29,481 (in 2018 dollars, 2014–2018) [72]. Approximately 25% of respondents had children in the household (*SD* = 0.43), while 30% of households in Detroit have children under 18 [73]. Average household size of the sample is 2.7 (*SD* = 1.38) persons, which closely matches Detroit at 2.6 persons (2014–2018). Socio-demographics were included in the analysis as independent variables. The data can be found in the supporting information section.

### 4.2 Measuring growth of produce in urban settings

Growing fruit and vegetables at home and in urban community gardens served as dependent variables. First, the following information about community gardens was provided:

*Community gardens are plots of urban land on which community members can grow flowers or foodstuffs (e.g., fruits and vegetables) for personal or collective benefit. Community gardeners share certain resources, such as space, tools, and water. Though often facilitated by social service agencies, nonprofit organizations, park and recreation departments, housing authorities, apartment complexes, block associations, or grassroots associations, community gardens nevertheless tend to remain under the control of the gardeners themselves*.

Respondents were asked the following questions: (1) Are you currently growing fruits in a community garden? (2) Are you currently growing vegetables in a community garden? (3) Are you currently growing fruits at home? (4) Are you currently growing vegetables at home? The answer categories ranged from 0 = never to 4 = always. In the subsequent analysis, answers for fruits and vegetables were combined into “produce” (summed and then divided by two). Since categories one through four had few answers compared to the zero category, values greater than zero were recoded as one. I used two binary dependent variables, one for growing produce in home gardens and one for community gardens: 0 = not growing and 1 = growing.

### 4.3 Measuring trust

The concept of generalized trust, as used in Grebitus et al. [74], was applied. It was measured using an instrument from the Generalized Social Survey, as follows: “Generally speaking, would you say that most people can be trusted or that you should be very careful in dealing with people?” This is a commonly used question [75]. Respondents chose between “yes,” “no,” and “I don’t know.”

### 4.4 Knowledge and attitude scale

Knowledge and attitude regarding the growing of food were measured using a bipolar 7-point scale based on Joiner [76], with items such as “I am very positive/negative about growing food” (see Table 3). Exploratory factor analysis, that is, principal component analysis with varimax rotational strategy, combined highly correlated items into independent factors. Factor reliability was measured using Cronbach’s alpha, which should be greater than 0.5 to retain a factor [77, 78].

### 4.5 Involvement scale

The New Involvement Profile (NIP) [79] was used to measure involvement in food-related topics [59, 80]. It comprises five involvement dimensions: (1) *relevance* addresses the importance of an activity; (2) *pleasure*, the amount of joy an activity brings; (3) *sign*, the prestige of an activity; (4) *risk importance*, the possible risk of an activity; (5) *risk probability*, how an activity’s risk (potential negative consequences) is perceived [59]. Participants evaluated each NIP item on a 7-point bipolar scale [79], choosing items that best described how they perceived growing food at home or in community gardens. Items included corresponding terms, such as “When growing food, I am certain/uncertain of my actions” (see Table 4). NIP data were analyzed using factor analysis, as above.

### 4.6 Personality scale

Personality was measured using the Midlife Development Inventory (MIDI) [63], which has been used to investigate the effect of personality on the acceptance of GM pork [67], demand for organic produce [66], preference and willingness to pay for organic and local applesauce [81], and willingness to pay for food miles [65]. MIDI measures personality traits through adjectives participants evaluate from 1 (not at all) to 4 (a lot), indicating how well they feel the adjectives describe them. The traits conscientiousness, agency, neuroticism, agreeableness, openness, and extraversion were derived by summing up related adjectives and dividing the sum by the number of adjectives.

### 4.7 Bivariate probit model

Since there were two dependent variables, a bivariate probit model was estimated in STATA 14 to jointly model produce growth. This model estimates the likelihood of growing produce both at home or in a community garden simultaneously, as they might influence each other: Someone growing produce at home might be more or less likely to grow produce in a community garden, and vice versa. A significant positive or negative correlation coefficient would indicate cross-equation gardening effects, so that gardening at each location is not independent. The correlation coefficient rho (*ρ* provides a measure of whether two single probit models or one bivariate probit model is sufficient [82]; If *ρ* is significant, the bivariate model is preferred. In summary, the bivariate probit model estimates home and community gardening jointly and tests whether the two equations are independent.

The binary variables identifying gardening were created as follows:

$$y_{i,home}=y_{i1}=\left\{ \begin{array}{c} 1,\;if\;participant\;is\;home\;gardening\\ 0,\;otherwise \end{array}\right.$$ (1)

$$y_{i,commga}=y_{i2}=\left\{ \begin{array}{c} 1,\;if\;participant\;is\;community\;gardening\\ 0,\;otherwise \end{array}\right.$$ (2)

where *home* refers to home gardening and *commga* to community gardening. The interdependency of gardening at home and community gardens is analyzed by estimating a bivariate probit model (see Greene [83]), which involves estimating the following two equations:

$$y_{i1}^{*}=\beta_{1}^{\prime }x_{i1}+\varepsilon_{i1},\;y_{i1}=1\;if\;y_{i1}^{*}>0,\;0\;otherwise,$$ (3)

$$y_{i2}^{*}=\beta_{2}^{\prime }x_{i2}+\varepsilon_{i2},\;y_{i2}=1\;if\;y_{i2}^{*}>0,\;0\;otherwise,$$

where *y*_*i*1_ and *y*_*i*2_ are binary variables representing individual observations, defined in Eq (1) and Eq (2); $\beta_{1}^{\prime }$ and $\beta_{2}^{\prime }$ are vectors of coefficients associated with ***x*_*i*1_** and ***x*_*i*2_** sets of explanatory covariates, and *ε*_*i*1_ and *ε*_*i*2_ are error terms. The explanatory covariates included are based on behavioral and socio-demographic variables from the survey. The equation takes the following form for home and community gardening:

$$\begin{array}{l} {Gardening}_{1,2}^{*}=\beta_{0}+\beta_{1}Trust_{i}+\beta_{2}Knowledge_{i}+\beta_{3}Attitude_{i}+\beta_{4}Relevance_{i}+\beta_{5}Pleasure_{i}+\beta_{6}\\ RiskProbability_{i}+\beta_{7}Agency_{i}+\beta_{8}Agreeableness_{i}+\beta_{9}Openness_{i}+\beta_{10}Neuroticism_{i}+\beta_{11}\\ Extraversion_{i}+\beta_{12}Conscientiousness_{i}+\beta_{13}Female_{i}+\beta_{14}Age_{i}+\beta_{15}Household\_size_{i}+\beta_{16}\\ Children_{i}+\beta_{17}Education_{i}+\beta_{18}Income_{i}+\beta_{19}White_{i}+\beta_{20}Black_{i}+\beta_{21}Native_{i}+\beta_{22}Asian_{i}+\varepsilon_{i}, \end{array}$$ (4)

where *Trust* is a binary variable equal to one if the individual is generally trusting; *Knowledge* and *Attitude* are factor scores for knowledge and attitude toward growing food; *Relevance*, *Pleasure*, and *RiskProbability* are involvement measures; *Agency*, *Agreeableness*, *Openness*, *Neuroticism*, *Extraversion*, and *Conscientiousness* are personality trait measures; *Female* is a dummy variable equal to one if the respondent is female; *Age* (*years*) is a continuous variable; *Household_size* is a continuous variable indicating how many people live in the respondent’s household, including the respondent; *Children* is a dummy variable equal to one if the respondent has children in the household; *Education* is an ordinal variable ranging from 1 (lowest) to 7 (highest); *Income* is an ordinal variable ranging from 1 (lowest) to 12 (highest), indicating approximate annual household income before taxes; *White*, *Black*, *Native*, and *Asian* are dummy variables equal to one if the respondent self-identified as White, Black or African American, American Indian or Alaska Native, or Asian, respectively; and ε_i_ is the unobserved random error term.

## 5. Empirical results

### 5.1 Growing produce in urban settings

Table 1 displays the results for growing produce at home and in community gardens. The results show that about 50% of the sample never grow fruit at home and 34% never grow vegetables at home. Approximately 70% and 66% never grow fruits and vegetables, respectively, in community gardens. From 0 = never to 4 = always, growing fruits at home had a mean of *M* = 1.27 (*SD* = 1.51), and growing vegetables at home had a mean of *M* = 1.81 (*SD* = 1.62). Growing fruits in community gardens had a mean of *M* = 0.82 (*SD* = 1.38), and growing vegetables in community gardens had a mean of *M* = 0.93 (*SD* = 1.45).

**Table 1:** Growing fruits and vegetables.

	Home garden				Community garden			
	Fruits		Vegetables		Fruits		Vegetables	
	Freq.	Percent	Freq.	Percent	Freq.	Percent	Freq.	Percent
Never	201	47.86	143	34.05	290	69.5	276	65.87
Sometimes	73	17.38	63	15.00	29	6.90	30	7.16
About half the time	43	10.24	44	10.48	23	5.48	25	5.97
Most of the time	37	8.81	69	16.43	41	9.76	43	10.26
Always	66	15.71	101	24.05	37	8.81	45	10.74

For the subsequent analysis, the data for fruits and vegetables were combined into *produce*, showing that 67% grew produce at home at least sometimes and 35% grew produce in community gardens at least sometimes. These two variables served as dependent variables in the subsequent bivariate probit models. Table 2 provides a cross-tabulation with these variables to show who grows at home, in the community garden, or in both locations. The results showed that 31% did not garden at either location, while 34% grew produce at home, 2% grew produce in the community garden, and 33% grew produce at both. The latter is noteworthy given that it indicates that those who are interested in growing produce are more likely to make use of multiple urban settings than those who are less interested. The results suggest that growing at home and in community gardens are likely to be correlated, making the bivariate probit model appropriate.

**Table 2:** Correlation analysis of home and community gardening.

Grow produce at community garden	Grow produce at home garden		
	Never	At least sometimes	Total
Never	31%	34%	65%
At least sometimes	2%	33%	35%
Total	33%	67%	100%

The following descriptive results will be presented for the full sample and separately for gardeners and non-gardeners based on the correlation analysis. Non-gardeners will be specified as those never growing produce (31%), and gardeners will be the remainder. These statistics were not broken down by home vs. community gardening, given that only 2% garden exclusively in community gardens.

### 5.2 Personal characteristics of gardeners and non-gardeners

Gardeners and non-gardeners differ to some extent in their personal characteristics. Of those who gardened, only 16% live in single households (29% couples), but of those who do not garden, 29% live in single households (37% couples). Of those who garden, 29% have children in the household as compared to those who do not garden (17%). Of those who garden, 75% are White as compared to those who do not garden (72%). Black/African American participants show a reverse picture, accounting for 16% of those who garden and 23% of those who do not. Those who garden have a disproportionately high share of lower education. For details, see S1 Table.

### 5.3 Trust

Regarding trust, the majority of participants felt that “you should be very careful in dealing with people” (54%). About 40% said that “most people can be trusted,” and the remaining 5% answered “I don’t know.” Analyzing the differences in trust between gardeners and non-gardeners revealed that 46% of gardeners are trusting, but only 31% of non-gardeners. In the following analysis, “most people can be trusted” was coded equal to one (41%) and the other responses equal to zero (59%).

### 5.4 Knowledge and attitude

Table 3 shows the mean for subjective knowledge and attitudes toward growing food for the total sample, gardeners, and non-gardeners. Participants agreed most with the statements that growing food was excellent and desirable and that they feel positive about it. However, they indicated that they did not have a great deal of experience growing food and did not consider it a favorite activity. Gardeners scored higher on positive statements than non-gardeners. The midpoint of the scale is four, meaning that scores below four are in agreement with negative statements, for example, “I dislike growing food very much.” Answers below this midpoint were found for five out of the eight statements for non-gardeners. They agreed slightly with statements such as being unfamiliar with growing food, having no experience with it, and that it is their least favorite activity. Between the two groups, agreement with knowledge statements (Factor 1) differed more than agreement with attitude statements (Factor 2).

**Table 3:** Knowledge and attitude regarding food growing.

Mean		Total sample	Gardener	Non-gardener	Factor
I have had a lot of exposure to growing food	I have had no exposure to growing food	4.37	4.88	3.25	1
I am extremely familiar with growing food	I am extremely unfamiliar with growing food	4.38	4.98	3.05	1
I have had a great deal of experience with growing food	I have had no experience with growing food	4.18	4.75	2.92	1
Growing food is my favorite activity	Growing food is my least favorite activity	3.94	4.51	2.70	1
Growing food is excellent	Growing food is poor	5.68	5.86	5.31	2
Growing food is desirable	Growing food is undesirable	5.48	5.68	5.05	2
I am very positive about growing food	I am very negative about growing food	5.38	5.70	4.69	2
I like growing food very much	I dislike growing food very much	4.83	5.31	3.77	2

*Note*: A bipolar scale with seven points was used. An answer of 7 indicated full agreement with statements on the left, and 1 indicated full agreement with statements on the right.

The data were analyzed using exploratory factor analysis. The rotated component matrix is presented in S2 Table. The Kaiser–Meyer–Olkin (KMO) criterion was 0.88 (meritorious).

The following factors were found:

#### 5.4.1 Factor 1: Knowledge and experience regarding growing food

This factor contained items related to knowledge and experience, indicating that one is familiar with growing food, has a lot of experience and exposure to growing food, and that growing food is a favored activity. The Cronbach’s alpha was 0.9188 (excellent).

#### 5.4.2 Factor 2: Attitude regarding growing food

This factor summed-up statements that express attitudes, such as being positive about growing food, and included opinions that growing food is excellent and desirable. The Cronbach’s alpha was 0.8908 (good).

### 5.5 Involvement

To measure involvement in the growing of food, respondents completed Jain and Srinivasan’s [79] involvement inventory (see Table 4). The results show that respondents thought growing food was beneficial, needed, and fun. Regarding the involvement dimensions, individuals were most involved with the dimensions *Relevance* and *Pleasure* and least involved with *Risk Probability*. Gardeners and non-gardeners differed most on the dimension of *Risk Probability*, wherein non-gardeners were less certain about how to grow, and the dimension of *Pleasure*, wherein growers were more excited about gardening. Both groups had the highest mutual agreement with regard to *Relevance*, and believed that growing food is essential, beneficial, and needed.

**Table 4:** Involvement with growing food.

Involvement dimensions (Mean)		Total sample	Gardeners	Non-gardeners
**Relevance**		5.37	5.41	5.28
Growing food is essential. /	Growing food is not essential	5.09	5.12	5.02
Growing food is beneficial /	Growing food is not beneficial	5.69	5.74	5.58
Growing food is needed /	Growing food is not needed	5.34	5.38	5.25
**Cronbach’s alpha = 0.67 (questionable)**				
**Pleasure**		4.89	5.14	4.33
I find growing food pleasurable /	I do not find growing food pleasurable	4.76	5.05	4.12
Growing food is exciting /	Growing food is unexciting	4.87	5.05	4.46
Growing food is fun /	Growing food is not fun	5.04	5.32	4.42
**Cronbach’s alpha = 0.75 (acceptable)**				
**Sign**		3.85	4.00	3.52
Growing food tells others about me /	Growing food doesn’t tell others about me	4.3	4.47	3.92
Others use me growing food to judge me /	Others won’t use me growing food to judge me	3.01	3.14	2.73
Growing food portrays an image of me to others /	Growing food does not portray an image of me to others	4.25	4.39	3.92
**Cronbach’s alpha = 0.24 (unacceptable)**				
**Risk importance**		4.24	4.16	4.39
It is really annoying to grow food that turns out unsuitable /	It is not annoying to grow food that turns out unsuitable	4.44	4.33	4.68
A poor turnout (low yield) when growing food would be upsetting /	A poor turnout (low yield) when growing food would not be upsetting	4.4	4.34	4.54
A lot to lose by poor turnout (low yield) when growing food /	Little to lose by poor turnout (low yield) when growing food	3.87	3.82	3.96
**Cronbach’s alpha = 0.31 (unacceptable)**				
**Risk Probability**		3.53	3.27	4.11
When growing food, I am uncertain of my actions /	When growing food, I am certain of my actions	3.48	3.14	4.22
I never know if I am doing the right thing when growing food /	I know for sure that I am doing the right thing when growing food	3.74	3.48	4.29
I feel a bit at a loss when growing food /	I don’t feel at a loss when growing food	3.39	3.19	3.83
**Cronbach’s alpha = 0.65 (questionable)**				
**KMO = 0.80 (meritorious)**				

*Note*: Bivariate scale with seven points were used, wherein 7 indicates full agreement with left-hand side statements, and one indicates full agreement with right-hand side statements.

Factor analysis was applied following Jain and Srinivasan [79] to analyze the five dimensions of involvement. Based on the KMO criterion, the validity of the items was meritorious. Cronbach’s alpha varied by factor (Table 4). The dimensions *Sign* and *Risk Importance* had unacceptable Cronbach’s alphas and thus were not included as variables in subsequent analysis. The reason for this Cronbach’s alpha result could be due to the fact that about one-third of the sample respondents did not garden; this could especially affect items that might be more relevant for urban agriculture participants. In addition, the factor analysis presented a four-factor solution instead of the five-factor solution in Jain and Srinivasan [79], and the factors did not resemble the original factors. Therefore, we followed Drescher et al. [59] and used an index (mean) to transform the values into indexes related to the five original NIP dimensions. This has the advantage that the indexes used in the subsequent analysis can be interpreted as Jain and Srinivasan’s [79] involvement dimensions. The indexes were normalized before inclusion in the subsequent analysis.

### 5.6 Personality

The results for personality showed that conscientiousness (*M* = 3.3), agreeableness (*M* = 3.3), and openness (*M* = 3.0) were the strongest traits, followed by extraversion (*M* = 2.9), agency (*M* = 2.6), and neuroticism (*M* = 2.3). Differences between gardeners and non-gardeners were very small, ranging between 0.01 and 0.21 (see S3 Table). For subsequent analysis, the six personality traits were normalized by subtracting the sample mean before including them in the bivariate probit models.

### 5.7 Socio-behavioral drivers of home and community gardening

The subsequent empirical analysis investigated the behavioral (psychological) and socio-demographic (personal) drivers of growing produce at home and in community gardens. Table 5 presents the results from the bivariate probit models. To understand the influence of individual factors, single models were tested before the comprehensive full model was tested. Results for trust, knowledge, and attitude were robust, in that the size, signs, and significance of the coefficients did not vary between models. All involvement dimensions were significant when modeled separately, but only *Relevance* remained significant once other variables entered the model. The same was true for personality, albeit to a lesser extent. Socio-demographic effects were robust, except for education and income.

**Table 5:** Drivers of home and community gardening.

	Model 1				Model 2				Model 3					Model 4				Model 5				Model 6 (full model)			
At	home		CG		home		CG		home		CG			home		CG		home		CG		home		CG	
Grow produce	Coef.		Coef.		Coef.		Coef.		Coef.		Coef.			Coef.		Coef.		Coef.		Coef.		Coef.		Coef.	
Trust	0.397	***	0.148																			0.327	**	0.126	
Knowledge (F)					0.655	***	0.527	***														0.694	***	0.489	***
Attitude (F)					0.177	**	-0.093															0.172	*	0.001	
Relevance (I)									-0.108	*	-0.217		***									-0.059		-0.157	**
Pleasure (I)									0.150	***	0.111		*									0.094		0.108	
Risk probability (I)									-0.185	***	-0.117		**									0.043		0.043	
Agency (P)														0.012		0.137						0.158		0.161	
Agreeableness (P)														-0.163		-0.243	*					-0.060		-0.087	
Openness (P)														0.098		0.099						-0.216		-0.078	
Neuroticism (P)														-0.117		-0.134						-0.084		-0.225	*
Extraversion (P)														0.298	**	0.481	***					0.064		0.293	*
Conscientiousness (P)														-0.161		-0.455	***					-0.001		-0.230	
Female																		-0.092		-0.443	***	0.023		-0.268	*
Age in years																		-0.004		-0.014	***	-0.008		-0.015	***
Household size																		0.131	**	0.086		0.149	**	0.097	
Children in HH																		0.182		0.257		0.036		0.172	
Education																		-0.057		-0.096	*	-0.002		-0.056	
Income																		0.008		-0.036		0.007		-0.047	*
White																		0.026		-0.335		0.135		-0.239	
Black or African American																		-0.296		-0.084		-0.337		-0.072	
American Indian or Alaska Native																		-0.211		0.476		0.001		0.602	
Asian																		0.351		0.034		0.154		0.029	
Constant	0.288	***	-0.451	***	0.522	***	-0.449	***	0.961	**	0.629			0.458	***	-0.415	***	0.482		0.969	**	0.309		0.613	
LR test of rho = 0: chi^2^	69.67				42.53				58.42					63.51				64.51				44.25			
Prob > chi^2^	0				0				0					0				0				0			
Wald chi^2^	9.15				111.3				43.57					38.81				168.70				213.94			
LP LL	-483.3				-429.6				-465.6					-464.4				-437.4				-367.9			
Prob > chi^2^	0.01				0				0					0				0				0			

Note: LP LL = log pseudo-likelihood. CG = community garden; (F) = factor; (I) = involvement; (P) = personality.

* = p<0.1

** = p<0.05

*** = p<0.01.

On analyzing the value of likelihood, the full model had a better model fit than the individual models. The correlation coefficient rho was 0.710 (*p* = 0.066), suggesting that growing produce at home and in community gardens were positively correlated. This confirms the descriptive findings and indicates that those who were likely to grow produce at home were also likely to grow produce in the community garden, and vice versa. This result was supported by the Wald test.

Results from the full model for psychological factors showed that trusting individuals were more likely to grow produce at home. The hypothesis that trust affects community gardening has not yet been confirmed. Being more knowledgeable increases the likelihood of growing produce at home or in community gardens. Having a positive attitude toward growing food only increased the likelihood of growing produce at home. In the full model, only one of the involvement dimensions affected participation in urban agriculture. *Relevance* had a significant negative effect on the growing of produce in community gardens. Given the measurement scale, this indicates that those who thought growing food was essential, beneficial, and needed were more likely to grow produce in the community garden. In the full model, an individual’s personality had no effect on growing produce at home. However, those who were extraverts were more likely to grow produce in community gardens. The more neurotic the respondents, the less likely they were to grow produce in community gardens.

In addition to the effects of psychological factors, personal factors influenced gardening. Compared to men, women were less likely to grow produce in community gardens, and the same held for older as compared to younger individuals. The bigger the household, the more likely the respondents were to grow produce at home, as suggested by a statistically significant and positive coefficient. A higher income reduced the likelihood of growing produce in community gardens.

## 6. Discussion

Agriculture in urban spaces has multiple benefits. Small-scale urban agriculture is related to increased overall health and well-being, nourishing individuals by providing healthy food options and building communities. This research developed a conceptual framework to highlight the socio-behavioral factors that drive the growing of produce in urban settings.

### 6.1 Descriptive findings

The findings of this study showed that at home, more than 15% of the participants always grew produce, but a large share of over 40% never grew produce. These findings complement the literature; for example, Kortright and Wakefield [42] found that more than 50% of households in Toronto grew food, and two-thirds of New Zealand secondary school students had a home vegetable garden [44]. Since a larger share of survey respondents did not grow produce in community gardens, this could explain why they felt less knowledgeable about growing food. Given the benefits of food gardening, increasing knowledge might increase participation in urban agriculture [42, 53]. Furthermore, participants had a generally positive attitude toward growing produce, and they were most involved with the *Relevance* and *Pleasure* provided by urban agriculture. The majority of respondents indicated that they had low levels of trust, conscientiousness, and agreeableness.

### 6.2 Differences between gardeners and non-gardeners

Some differences with regard to socio-demographics were noted between gardeners and non-gardeners. Gardeners were characterized as living in larger households with children and having lower education level. Furthermore, they seemed to be more trusting (46%, compared to 31% for non-gardeners). This could mean that non-gardeners do not grow produce because they do not trust themselves to know how to grow produce or that they do not want to join a community garden because they do not trust others. Moreover, trust has been found to be high among community gardeners [26]; hence, increasing trust among non-gardeners could encourage them to start gardening. Looking deeper into the motives for distrust could be an avenue for future research.

Gardeners and non-gardeners differed in their knowledge and attitude toward growing food, and the difference was more pronounced for knowledge, which is likely related to gardeners being more experienced than non-gardeners. With regard to involvement dimensions, differences were found especially for *Risk Probability*, wherein non-gardeners were more uncertain about how to grow, and *Pleasure*, wherein gardeners displayed more excitement. Both groups were in agreement with regard to the *Relevance* of growing food. Differences in personality between gardeners and non-gardeners were small.

### 6.3 Behavioral factors influencing home and community gardening

Econometric analysis showed that several factors influenced the likelihood of growing produce at home and in community gardens. Those who were more trusting were more likely to grow produce at home, but contrary to the initial hypothesis, trust had no effect on growing produce in community gardens. This could be explained by home gardening, which requires individuals to trust themselves to grow food. According to Kopiyawattage et al. [52], having trust in one’s own abilities can be related to perceived behavioral control, which in turn influences decisions for urban farming.

It was tested whether subjective knowledge affects participation in urban agriculture. The analysis showed that respondents who felt more knowledgeable about growing food were more likely to garden at home and in community gardens. This is in line with previous research showing that subjective knowledge affects behavior related to ecological footprints [84], recycling [85], participation in commercial urban agriculture [3], and decisions to continue farming in urban areas [52]. The data also showed that a positive attitude toward urban food growing increased the likelihood of growing food at home. This is in line with Grebitus et al. [3]. who found that a generally positive attitude toward urban agriculture increased the likelihood of participating in commercial urban agriculture. Kopiyawattage et al. [52] showed that for commercial urban food producers, positive attitudes affected the decision to continue farming. The lack of effect on community gardening might be explained by community gardens serving several purposes in addition to growing food.

The involvement dimension *Relevance* was significant for growing food in community gardens, indicating that thinking produce growing is essential, beneficial, and needed increases the likelihood of growing produce. This is in line with past research showing that *Relevance* was the most important involvement dimension with regard to food product choices [59]. The findings showed that extraverted personalities were more likely to grow produce in community gardens; this makes sense intuitively, given that community gardens cater to these individuals’ outgoing nature, allowing them to have more social interactions. At the same time, neuroticism was significant and negative for growing produce in community gardens. Winter and Grebitus [86] found that the same traits had significant effects on private food label choices.

### 6.4 Socio-demographic factors influencing home and community gardening

With regard to socio-demographics, respondents with higher income were less likely to grow produce in community gardens. These findings contrast with those of Bellemare and Dusoruth [69], who found that respondents in Montreal with less than C$20,000 income were less likely to practice urban agriculture, although the finding was not differentiated for home and community gardening. Comparing home and community gardeners in San Jose, Algert et al. [45] found that community gardeners have higher income and higher education. Furthermore, results showed that female participants were less likely to grow produce in community gardens, similar to Van Lier et al. [44], who found that male adolescents in New Zealand were more likely to participate in home gardening. These findings differ from those of Bellemare and Dusoruth [69], who found that men were less likely than women to be urban gardeners, and those of Grebitus et al. [3], who showed that female individuals were more likely to grow food at an urban farm. However, Grebitus et al. [3] used a hypothetical setting to study urban agriculture participation in Phoenix, where respondents were asked about their likelihood to garden rather than their actual gardening. Moreover, results indicated that older individuals were less likely to grow produce in community gardens, similar to Bellemare and Dusoruth [69] and Van Lier et al. [44], who found that younger individuals were more likely to participate in urban agriculture. However, these findings contrast with those of Grebitus et al. [3], who showed that older individuals were more likely to grow food at an urban farm, albeit in a hypothetical setting. Algert et al. [45] also found community gardeners to be older than non-gardeners.

The larger the household, the more likely the respondents were to grow produce at home, probably because larger households are more likely to have a house with a yard where growing food is more feasible. These results are in line with Bellemare and Dusoruth [69] who also found that larger households were more likely to participate in urban agriculture.

Overall, the findings reflect that studies in different regions with different populations have different results for personal factors. Furthermore, whether studies differentiate between growing produce at home and in community gardens affected the findings.

### 6.5 Research limitations

This study has some limitations. To measure the constructs, a survey with direct, self-reported measures was used, which may have resulted in social desirability bias [87]. However, online surveys result in lower bias than, for instance, telephone interviews [88]; hence, the social desirability bias in this case can be considered minimal. Nonetheless, using an online survey can cause other concerns, such as self-selection bias [89]; this risk was countered by using a relatively large sample from a local area. Moreover, coverage can bias findings; for example, under-coverage is possible, given that specific groups may be under-represented compared to the overall US population. This is the case when specific groups in the population are not as well represented by the sample, for example those who have less access to the Internet [89]. Such coverage problems might have affected this study, because in Detroit, only 80% of all households have a computer as compared to the U.S. as a whole, wherein 89% of all households have a computer. Further, fewer households in Detroit (59%) have a home Internet connection compared to the U.S. in total (80%) [71]. This needs to be considered when drawing conclusions and could be addressed by future research collecting data from other regions of the U.S. to test if the results for Detroit are generalizable. In addition, it could be of value to study regions outside the U.S. and compare findings to the present study. More generally, the sample does not represent Detroit in terms of race; hence, the findings are not generalizable across the study region, but need to be interpreted in the context of the socio-demographics of the sample. Finally, many factors affect the growing of food. Resources are another driver that influences the decision to garden. This study is limited in that it focused on socio-behavioral factors, without including resources other than income. Future research could extend the present study’s framework by including variables on resources, such as space and time.

### 6.6 Suggestions for future research

As mentioned in the limitations, the factors that influence the growing of produce at home or in community gardens are manifold. Future research could include other determinants, such as time constraints, mobility, transportation, financial resources, space availability at home, and access to community gardens to shed more light on barriers to urban farming. Further analysis could also investigate interactions between physical resources (time, money, space) and behavior. For example, in studying urban agriculture in California, Surls et al. [90] analyzed what commercial urban farmers need when faced with limited resources. They showed that land access, long-term availability, production issues, regulations, and business planning/marketing are some of the challenges. Moreover, land access and long-term availability are roadblocks that community gardens have encountered, and similar barriers were found by Schupp et al. [91] regarding home gardening in Ohio. They found that income, education, space availability, and housing type determined whether households participated in home gardening. Future research could supplement this analysis.

## 7. Conclusion

This research aimed to close a gap in the literature: the lack of quantitative research that focuses on the socio-behavioral drivers of home and community gardening. A conceptual framework was tested, and the results indicated that knowledge affects growing produce both at home and in community gardens. Other factors differed for growing produce at home and in community gardens. For example, involvement and personality only affected growing produce in community gardens, while trust only affected home gardening. Socio-demographics also affected the growing of produce.

Given the benefits, urban farming seems promising, and this study has several implications for promoting small-scale urban agriculture. The first, which is relevant to nutrition education and university extension services, is that increased knowledge leads to increased participation in home and community gardens. Hence, we need to educate future gardeners, to increase their knowledge and ability to participate safely in small-scale urban agriculture, as stressed by Kortright and Wakefield [42], who suggested that home food gardeners could be supported with regard to acquiring ecological gardening skills and to general learning opportunities. Lack of knowledge can increase the risk for those who are unaware of safe gardening practices, for example the risk of soil contaminants. In addition, home gardeners might cause nutrient loading of stormwater runoff in urban areas due to the overuse of chemical fertilizers and pesticides [40]. Hence, Sanye-Mengual et al. [43] recommended minimizing the use of chemicals, integrating pest management, making use of renewable resources, and diffusing nursing. Taylor and Taylor Lovell [40] called for outreach and research to train gardeners in safe and sustainable food growing practices that support ecosystem health. This could include NGOs being more involved in small-scale urban agriculture through education, outreach, and research programs.

Given that growing food at home is affected by trust, peoples’ trust in their gardening abilities needs to be improved, ideally through education and by training them early. This was pointed out by Landry et al. [53], who recommended including gardening in the school curriculum to build skills and increase the probability of maintaining community gardens. Given that I found that a positive attitude increased the likelihood of participating in home food gardening only, another implication for community gardens is to introduce strategies in their recruitment efforts that improve attitudes toward growing food.

Because involvement (*Relevance*) affects home gardening, conveying to non-participants that growing food is essential and beneficial might increase their participation. Results for personality showed that extraversion and neuroticism, in particular, determined participation in small-scale urban agriculture. This suggests that these two personality traits are important in explaining behavior, and hence should be considered when targeting education activities and designing community gardens. For instance, extraverts are already open to participating in urban agriculture, but neurotic personas may feel uncomfortable. Programs can be designed in a way that alleviates the anxiety of neurotic personas; given that gardening has been shown to help with depression, mental well-being, and health, this could be especially beneficial.

Extension services and NGOs already offer gardening education and outreach, therefore, the main recommendation of this study is to expand and strengthen the programs that are already in place (see Beavers et al. [92] regarding gardening support programs in Detroit). In conclusion, the conceptual framework developed in this study and the significant effects of the socio-behavioral factors identified can be utilized to derive target-oriented educational strategies to help community gardens, NGOs, university extension, and other stakeholders increase participation in small-scale urban agriculture.

## Supporting information

S1 Table Socio-demographic characteristics of gardeners and non-gardeners. (DOCX)

S2 Table Knowledge and attitude regarding food growing. (DOCX)

S3 Table Personality traits. (DOCX)
