# Supplementary material for: Small-scale urban agriculture: Drivers of growing produce at home and in community gardens in Detroit
Source: PLoS One. 2021 Sep 7;16(9):e0256913. doi: 10.1371/journal.pone.0256913 (PMC8423299; doi:10.1371/journal.pone.0256913)
Supplement: S1 Table — (DOCX) [file pone.0256913.s001.docx]

| **In %** |  | **Sample** | **Gardeners** | **Non-gardeners** |
| --- | --- | --- | --- | --- |
| **Gender** | Male | 50 | 51 | 48 |
|  | Female | 50 | 49 | 52 |
| **Household** | 1 | 20 | 16 | 29 |
| **size** | 2 | 31 | 29 | 37 |
|  | 3 | 19 | 22 | 11 |
|  | 4 | 16 | 18 | 12 |
|  | 5 | 9 | 11 | 5 |
|  | 6 | 2 | 2 | 3 |
|  | 7 | 0 | 1 | 0 |
| **Children in** | No | 75 | 71 | 83 |
| **household** | Yes | 25 | 29 | 17 |
| **Age** | 18–24 | 13 | 14 | 12 |
| **in years** | 25–34 | 16 | 17 | 14 |
|  | 35–44 | 18 | 19 | 15 |
|  | 45–54 | 18 | 17 | 20 |
|  | 55–64 | 19 | 19 | 20 |
|  | 65–74 | 13 | 12 | 14 |
|  | 75–88 | 3 | 1 | 5 |
| **Race** | White | 74 | 75 | 72 |
|  | Black or African American | 18 | 16 | 23 |
|  | American Indian or Alaska Native | 1 | 1 | 2 |
|  | Asian | 4 | 5 | 3 |
|  | Native Hawaiian or Pacific Islander | 0 | 0 | 0 |
| **Education** | Less than high school | 2 | 3 | 2 |
|  | High school graduate | 23 | 24 | 19 |
|  | Some college | 27 | 27 | 27 |
|  | 2-year degree | 10 | 12 | 6 |
|  | 4-year degree | 26 | 24 | 31 |
|  | Professional degree | 9 | 8 | 13 |
|  | Doctorate | 2 | 2 | 3 |
| **Income** | Less than $10,000 | 8 | 8 | 10 |
|  | $10,000–$19,999 | 10 | 9 | 10 |
|  | $20,000–$29,999 | 12 | 12 | 11 |
|  | $30,000–$39,999 | 11 | 11 | 10 |
|  | $40,000–$49,999 | 10 | 8 | 12 |
|  | $50,000–$59,999 | 12 | 13 | 10 |
|  | $60,000–$69,999 | 6 | 7 | 5 |
|  | $70,000–$79,999 | 10 | 10 | 11 |
|  | $80,000–$89,999 | 4 | 4 | 4 |
|  | $90,000–$99,999 | 4 | 4 | 4 |
|  | $100,000–$149,999 | 10 | 11 | 7 |
|  | More than $150,000 | 4 | 3 | 6 |
