# Supplementary material for: Small-scale urban agriculture: Drivers of growing produce at home and in community gardens in Detroit
Source: PLoS One. 2021 Sep 7;16(9):e0256913. doi: 10.1371/journal.pone.0256913 (PMC8423299; doi:10.1371/journal.pone.0256913)
Supplement: S2 Table — (DOCX) [file pone.0256913.s002.docx]

| **Bipolar scale (left statement=7, right statement=1)** | | **F1 knowledge** | **F2 attitude** |
| --- | --- | --- | --- |
| Growing food is excellent | Growing food is poor | 0.1237 | 0.9087 |
| Growing food is desirable | Growing food is undesirable | 0.1920 | 0.8726 |
| I am very positive about growing food | I am very negative about growing food | 0.3279 | 0.8525 |
| I like growing food very much | I dislike growing food very much | 0.6231 | 0.5972 |
| I have had a lot of exposure to growing food | I have had no exposure to growing food | 0.8983 | 0.1904 |
| I am extremely familiar with growing food | I am extremely unfamiliar with growing food | 0.9091 | 0.2111 |
| I have had a great deal of experience with growing food | I have had no experience with growing food | 0.9276 | 0.1638 |
| Growing food is my favorite activity | Growing food is my least favorite activity | 0.6946 | 0.4183 |
| **Cronbach’s alpha** |  | 0.9188 | 0.8908 |
