# Supplementary material for: Small-scale urban agriculture: Drivers of growing produce at home and in community gardens in Detroit
Source: PLoS One. 2021 Sep 7;16(9):e0256913. doi: 10.1371/journal.pone.0256913 (PMC8423299; doi:10.1371/journal.pone.0256913)
Supplement: S3 Table — (DOCX) [file pone.0256913.s003.docx]

|  | **Total sample** | | **Gardener** | | **Non-gardener** | |
| --- | --- | --- | --- | --- | --- | --- |
|  | **M** | **SD** | **M** | **SD** | **M** | **SD** |
| Agency | 2.57 | 0.71 | 2.61 | 0.68 | 2.47 | 0.77 |
| Agreeableness | 3.30 | 0.64 | 3.31 | 0.65 | 3.27 | 0.61 |
| Openness | 3.03 | 0.57 | 3.06 | 0.56 | 2.96 | 0.60 |
| Neuroticism | 2.26 | 0.68 | 2.23 | 0.68 | 2.33 | 0.68 |
| Extraversion | 2.91 | 0.69 | 2.97 | 0.67 | 2.76 | 0.71 |
| Conscientiousness | 3.34 | 0.57 | 3.34 | 0.55 | 3.35 | 0.60 |
